# Brainstem-Evoked Transcription of Defensive Genes After Spinal Cord Injury

**DOI:** 10.3389/fncel.2019.00510

**Published:** 2019-11-19

**Authors:** Walter J. Jermakowicz, Melissa M. Carballosa-Gautam, Alberto A. Vitores, Ian D. Hentall

**Affiliations:** The Miami Project to Cure Paralysis, Department of Neurological Surgery, University of Miami, Miami, FL, United States

**Keywords:** spinal cord injury, mRNA, raphe magnus nucleus, electrical stimulation, rat (Brown Norway)

## Abstract

The spinal cord after injury shows altered transcription in numerous genes. We tested in a pilot study whether the nucleus raphé magnus, a descending serotonergic brainstem region whose stimulation improves recovery after incomplete spinal cord injury (SCI), can influence these transcriptional changes. Rats received 2 h of low-frequency electrical stimulation in the raphé magnus 3 days after an impact contusion at segment T8. Comparison groups lacked injuries or activated stimulators or both. Immediately following stimulation, spinal cords were extracted, their RNA transcriptome sequenced, and differential gene expression quantified. Confirming many previous studies, injury primarily increased inflammatory and immune transcripts and decreased those related to lipid and cholesterol synthesis and neuronal signaling. Stimulation plus injury, contrasted with injury alone, caused significant changes in 43 transcripts (39 increases, 4 decreases), all protein-coding. Injury itself decreased only four of these 43 transcripts, all reversed by stimulation, and increased none of them. The non-specific 5-HT7 receptor antagonist pimozide reversed 25 of the 43 changes. Stimulation in intact rats principally caused decreases in transcripts related to oxidative phosphorylation, none of which were altered by stimulation in injury. Gene ontology (biological process) annotations comparing stimulation with either no stimulation or pimozide treatment in injured rats highlighted defense responses to lipopolysaccharides and microorganisms, and also erythrocyte development and oxygen transport (possibly yielding cellular oxidant detoxification). Connectivity maps of human orthologous genes generated in the CLUE database of perturbagen-response transcriptional signatures showed that drug classes whose effects in injured rats most closely resembled stimulation without pimozide include peroxisome proliferator-activated receptor agonists and angiotensin receptor blockers, which are reportedly beneficial in SCI. Thus the initial transcriptional response of the injured spinal cord to raphé magnus stimulation is upregulation of genes that in various ways are mostly protective, some probably located in recently arrived myeloid cells.

## Introduction

Mechanical SCI causes numerous molecular, cellular and structural changes that vary in duration and onset, both at the site of injury and beyond (Dulin et al., 2015; O’Shea et al., 2017). These include changes in gene transcription that collectively and individually could influence recovery via protein synthesis and downstream molecules. It is therefore of interest to find ways to modify influential transcriptional changes by enhancing or opposing them according to their benefit or harm. Here we explore whether activity in a raphé nucleus of the hindbrain, the NRM, regulates transcription after SCI. The NRM is one of several ventral medullary nuclei that send overlapping serotonergic projections (as well as non-serotonergic projections) directly to the dorsal and ventral spinal cord, others being the nucleus raphe obscurus, nucleus raphe pallidus and the laterally adjacent paragigantocellularis and gigantocellularis nuclei (Kwiat and Basbaum, 1992; Gautier et al., 2017).

Previously, we showed that prolonged electrical stimulation of the NRM (for several days to weeks) significantly enhanced recovery from various autonomic, sensory, and motor deficits of contusional cervical or thoracic SCI in rats, while improving myelination and serotonergic innervation near the lesion (Hentall and Burns, 2009; Hentall and Gonzalez, 2012; Vitores et al., 2018). A shorter period of NRM stimulation (2 h) was found to restore injury-depleted levels in the spinal cord of cAMP and its downstream targets, phosphorylated cAMP response element-binding protein, and pPKA (Carballosa-Gonzalez et al., 2014), which cause neurotrophic effects via alterations in gene expression (Hannila and Filbin, 2008; Carballosa-Gonzalez et al., 2014; Batty et al., 2017). Stimulation of the midbrain’s median and dorsal raphé, which send ascending projections throughout the forebrain, analogously yielded partial restoration of anatomic and functional deficits following TBI in rats, as well as normalizing cAMP levels, thus establishing the generality of the idea that raphé nuclei provide central links for restorative feedback. In mice, prolonged NRM stimulation attenuated signs of experimental autoimmune encephalomyelitis, a model of MS, including cellular pathology and cytokine upregulation (Madsen et al., 2017), which further supports this idea. Our general aim in the studies cited above has been to achieve lasting, clinically significant recovery with a period of prolonged electrical DBS by evoking neurotrophic or protective effects from activated neuronal systems. In contrast, most studies of DBS for neurodegenerative conditions such as SCI, TBI, and MS have focused on the acute reversal of overt problematic signs or symptoms (e.g., pain, paralysis, altered mental status), without necessarily remedying the underlying pathology (Roy and Aziz, 2014; Shin et al., 2014; Chari et al., 2017).

Here, we examine transcriptional changes produced by NRM stimulation in rats with thoracic (T8) SCI. Stimulation was applied for 2 h to the NRM on the third day after a weight-drop SCI at segment T8. At this post-injury stage, many molecular pathways that promote plasticity and inflammation are near their peak (David and Kroner, 2011; Mao et al., 2016). The relatively brief period of stimulation, lasting just long enough for initial transcriptional effects to emerge, was followed by immediate extraction of various portions of the spinal cord and standard processing for RNA sequence analysis. Both injured and non-injured controls were studied with and without stimulation (*n* ≥ 3). Additional injured rats (*n* = 2) received stimulation after being pretreated with the non-specific 5-HT7 antagonist pimozide, to assess possible participation of serotonin release from the axon terminals of NRM neurons. The results proved surprising. Our working hypothesis was that stimulation in injured rats would induce neurotrophic transcriptional effects and predominantly reverse injury-produced changes. Instead it altered a small number of genes that are mostly concerned with inflammation and erythrocyte formation, very few of which were altered by injury alone. These findings shed new light on the brainstem’s descending modulatory influence on endogenous processes in healthy and injured spinal cord, and also identified molecules with potential for improving outcomes after SCI.

## Materials and Methods

### Animal Surgical Procedures and Treatments

All experiments were performed in accordance with the guidelines of the NIH Guide for the Care and Use of Laboratory Animals, and were approved by the University of Miami Miller School of Medicine Institutional Animal Care and Use Committee. Female young-adult Sprague-Dawley rats (220–240 g, 10–12 weeks old), obtained from Harlan Sprague-Dawley, Inc. (Indianapolis, IN, United States), were anesthetized with intraperitoneal ketamine (50 mg/kg) plus xylazine (10 mg/kg) and mounted in a stereotaxic head holder. Following a midline back incision and lateral dissection of the spinous musculature, a T8 laminectomy was performed and a moderate bilateral contusion injury was caused with a NYU-MASCIS Impactor, which applied a force by letting a 10 g rod of 2 mm diameter drop 12.5 mm centered on the midline of the spinal cord (Kearney et al., 1988). Some control animals received a laminectomy without the contusion injury. Dissected muscle layers were sutured and the skin was closed with wound clips. Animals recovered on a 37°C heating blanket. The opioid buprenorphine (0.01 mg/kg bid, subcutaneous) was given daily for analgesia and the antibiotic gentamycin (0.01 mg/kg bid, subcutaneous) to prevent infection. Bladder volume was checked daily and manually emptied.

At 72 h after the injury or sham injury, the animals were anesthetized with isoflurane (1.2% in oxygen) by face mask after induction in a glass chamber and mounted in a stereotaxic holder. A single monopolar stimulating microelectrode was placed in the brainstem by making a linear skin incision and drilling a 1.8 mm craniostomy on the midline, 2.2 mm caudal to the interaural line. To apply stimulation, a tungsten microelectrode (AC impedance 0.5 megohm, diameter 0.13 mm) was inserted into the midline NRM at the stereotaxic coordinates 2.2 mm caudal and 10 mm ventral to the interaural line (flat-skull orientation). Accuracy was confirmed by observing bilateral facial twitching 1.2–1.5 mm above the target, which disappeared once the electrode reached the NRM. This localization method was validated histologically in a previously published study (Hentall and Gonzalez, 2012). Monopolar stimulation, consisting of 8-Hz trains of cathodal pulses (30 μA, 1 ms), was given for 2 h in a 5 min, 50% duty cycle, ending in the stimulation phase. Electrodes were also placed in sham and injured control animals that did not receive the 2 h of stimulation. One injured group received pimozide by intraperitoneal injection of 1 mg/kg, 1 h prior to stimulation onset. Immediately after the stimulation period, the subdural spinal cord (including pia-arachnoid) was rapidly dissected and transversely sectioned into three 0.5 cm blocks encompassing the C4–C6, T7–T9, and L1–L3 spinal segments. The blocks were immediately placed in liquid nitrogen and stored at −80°C. Animals were euthanatized by isoflurane overdose. No adverse effects of stimulation or other treatment were seen.

### RNAseq Sample Preparation

Cervical, thoracic and lumbar regions were combined for RNA analysis. Preparation and sequencing of RNA libraries was carried out by personnel of the John P. Hussman Institute for Human Genomics at the Center for Genome Technology, University of Miami Miller School of Medicine. RNA isolated from rat spinal cords was extracted using Trizol and purified using the QIAGEN (Venlo, Netherlands) RNeasy kit, by following the kit’s standard protocol. Samples were analyzed with an Agilent Bioanalyzer (Santa Clara, CA, United States). All analyzed samples passed minimum quality and quantity threshold required for RNA sequencing, including RNA integrity score above 7.0. Mean concentration of total RNA were 0.43 μg/μl (*SD*: 0.15); the mean purity (260/280 ratio) was 2.06 (*SD*: 0.041) and was > 2.02 in all individuals. From each sample, 500 ng of total RNA was provided to an Illumina TruSeq Stranded Total RNA Library Prep Kit with Ribo-Zero (San Diego, CA, United States) to create ribosomal RNA-depleted sequencing libraries with a unique barcode. Sequencing was performed to >25 million raw reads in a single-end 75 bp sequencing run on the Illumina NextSeq500.

### Production of RNAseq Data

Raw sequence data was processed by the on-instrument Real Time Analysis software (v.2.7.7) transferred to de-multiplexed FASTQ files with the Illumina-supplied scripts in BCL2FASTQ software (v2.17). The quality of the reads was determined with FASTQC software^1^ for per base sequence quality, duplication rates, and overrepresented k-mers. Illumina sequencing adapters were trimmed from the ends of the reads using the Trim Galore! Package^2^, then aligned for *Rattus norvegicus* (Rnor_5.0) with the STAR aligner (v2.5.0a) (Dobin et al., 2013). Gene count quantification for aligned reads was performed using the GeneCounts function within STAR against the Ensembl gene build pipeline (release 79).

### Data Analysis

Experimental groups, to which rats were assigned in random order, were as follows: group A, sham injury and sham stimulation; group B, SCI and sham stimulation; group C, SCI and stimulation; group D, SCI with stimulation and pimozide; group E, sham injury with stimulation. Many subjects had also provided material for a previously published assay of cAMP, cAMP response element-binding protein and protein kinase A (Carballosa-Gonzalez et al., 2014); exceptions were one subject in group E and both subjects in group D, whose samples were contemporaneous with the rest but had not previously been assayed for publication. Within 5 experimental groups, there were 10 possible contrasts, of which 6 were analyzed (Table 1). These contrasts are named in the text by the positively changed factor followed in parentheses by the constant background. Thus, ±st(SCI) refers to the contrast of stimulation (st) with no stimulation in injured rats, ±SCI(st) contrasts injury with no injury in stimulated rats, ±pim(st/SCI) contrasts pimozide’s presence with its absence in stimulated, injured rats; ±st(0), ±SCI(0), and ±st/SCI(0) respectively contrast stimulation, injury or both with their absence (in rats with no other interventions).

**TABLE 1:** Groups and contrasts analyzed.

**Contrast: name, ratio**	**Group**	**Injury**	**Stimulation**	**Pimozide**	**Number flagged (up/down)**	**SD (difference)**
±SCI(0), B/A	B, *n* = 3	Yes	No	No	982(922/60)	1047
	A, *n* = 4	No	No	No		
±st(SCI), C/B	C, *n* = 3	Yes	Yes	No	43(39/4)	327
	B, *n* = 3	Yes	No	No		
±pim(st/SCI), D/C	D, *n* = 2	Yes	Yes	Yes	81(52/29)	601
	C, *n* = 3	Yes	Yes	No		
±st(0), E/A	E, *n* = 5	No	Yes	No	28(20/8)	226
	A, *n* = 4	No	No	No		
±st/SCI(0), C/A	C, *n* = 3	Yes	Yes	No	1150(1084/66)	854
	A, *n* = 4	No	No	No		
±SCI(st), C/E	C, *n* = 3	Yes	Yes	No	1374(1205/168)	911
	E, *n* = 5	No	Yes	No		

*The last column shows the numbers of significant differences in individual genes that were flagged by EdgeR software, according to the joint criteria FDR < 0.05 and abs(log_2_(FC)) > 1. The condition in the first row provided the numerator for FC, the second row provided the denominator. Increases [log_2_(FC) > 1] and decreases [0 < log_2_(FC) < 1] are consistently thus defined in this paper. The standard deviation (SD) was calculated from the differences in count taken over all genes. Underlining specifies the parameter(s) varied in the given contrast.*

Differential gene expression analysis was performed on the gene count data with edgeR software (Robinson et al., 2010). Gene counts were normalized against total aligned reads to generate counts-per-million for each gene in each sample. Given the relatively small sample sizes per group, the exact test implemented in EdgeR was used to determine differential expression, producing a false discovery rate (FDR) *p*-value. Significant differential expression was decided by EdgeR with joint criteria of FDR < 0.05 and a greater than 100% change: abs(log_2_(FC)) > 1, where FC is FC. For comparison, output from two other DGE software packages, DESeq2 and baySeq, was also examined (Hardcastle and Kelly, 2010; Love et al., 2014). Numbers of significant increases and decreases obtained in different contrasts were analyzed in 2 × 2 contingency tables with the two-tailed Chi-square test.

Pathway and network analyses were performed with Ingenuity Pathway Analysis (QIAGEN), using as input the lists of genes with FDR < 0.05, as determined by EdgeR. Gene ontology (biological process) analysis was performed for each set of genes meeting EdgeR cutoff criteria of abs(log_2_(FC)) > 1 and FDR < 0.05 with the DAVID^3^. Statistical overrepresentation was determined with the default settings in DAVID. Gene names used in tables were official gene symbols given by DAVID, supplemented by Gene from the United States’ NCBI.

Human orthologs of rat genes flagged in the various EdgeR contrasts were obtained from the Ensembl 2018 website (Zerbino et al., 2018). The resulting human gene list was applied to the L1000 gene expression database of the CLUE software platform (Subramanian et al., 2017). This platform produced a connectivity map that was analyzed with the Query tool to find classes of perturbagens (drugs) whose expression signatures most closely resembled those yielded by the experiments.

### Data Availability

The high-throughput sequence data is available from the GEO repository of the NCBI, accession number GSE133093. Other data relevant to the study’s conclusions are available on request from the corresponding author.

## Results

### Numbers Flagged as Upregulated or Downregulated

A total of 32,494 transcripts with unique rat gene identification (Ensembl) numbers were analyzed. These included 1,563 micro RNAs, 1,471 small RNAs, and 22,019 experimentally confirmed protein-coding RNAs; further details are given in the Supplementary Figure 1. For all six contrasts, the DGE software applications EdgeR and DESEq2 were of roughly equal leniency, and showed mean overlap of 75% for selected transcripts. The baySeq application was considerably more stringent, flagging on average 45% of those flagged by the other two software applications (Supplementary Figure 1). The following presentation is limited to results obtained with EdgeR. In the key contrasts ±st(SCI) and ±st(0), neither DESeq2 nor baySeq found any significant genes that were not flagged by edgeR.

The contrasts ±SCI(0), ±st/SCI(0) and ±SCI(st), all of which pair intact rats with rats injured 3 days previously, revealed a large number of differentially expressed transcripts. These numbers differed significantly (pairwise Chi-squared contingency tables, *p* < 0.0001). Contrast ±SCI(0) showed fewer differentially transcribed genes (*n* = 982) than ±st/SCI(0) (*n* = 1150), and ±SCI(st) showed the most differentially expressed transcripts (*n* = 1374). The relatively brief interventions of 2 h of stimulation or 3 h of pimozide’s presence caused far fewer significant alterations in transcription. The numbers of genes affected in the contrast ±pim(st/SCI) (*n* = 81) was significantly different (*p* = 0.0009) from the numbers for ±st(SCI) (*n* = 43), but the latter was not significantly different (*p* = 0.10) from the contrast ±st(0) (*n* = 28). With the brief interventions, there was also less variation in the difference between counts for the paired conditions, as reflected in their standard deviations (Table 1). All three interventions (injury, stimulation, pimozide) consistently produced more increases than decreases in transcript counts (Table 1): pimozide [contrast ±pim(st/SCI)] revealed 64% increases, injury alone [contrast ±SCI(0)] revealed 94% increases and stimulation after injury [contrast ±st(SCI)] revealed 91% increases. Differing kinetics of decay and synthesis may in some cases have contributed to this imbalance (see section “Discussion”).

### Genes Affected by Stimulation: Responses to Injury Alone

The contrast showing the effect of stimulation in injury, contrast ±st(SCI), was of greatest importance for the goals of this study. It found that 43 transcripts, all protein-coding, had changed significantly (Table 2). When one of these 43 transcripts was flagged in another contrast involving injury, the direction of the effect was consistent: for example, in the opposite direction of the effects from injury alone [contrast ±SCI(0)] and in the same direction as the effect of injury plus stimulation compared with untreated rats [±st/SCI(0)] and as the effect of injury versus sham on stimulated rats [±SCI(st)] (Table 2). However, it was notable that only 9% of injury-produced transcriptional changes [contrast ±SCI(0)], all downregulation, were reversed by NRM stimulation [contrast ±st(SCI)].

**TABLE 2:** Genes altered by stimulation in injured animals, selected by contrast ±st(SCI).

**Protein (gene)**	**±SCI(0)**	**±st(SCI)**	**±pim(st/SCI)**	**±st/SCI(0)**	**±SCI(st)**
^∗^Similar to 60S ribosomal protein L12 (*RGD1564883*)	–6.63	7.12	–10.44		
^∗^Cathepsin G (*Ctsg*)		3.90	–2.26		2.55
^∗^Carbonic anhydrase I (*Car1*)		3.78	–2.49		
^∗^Defensin NP-4 precursor (*Np4*)		3.69	–2.85		2.35
^∗^Matrix metallopeptidase 13 (*Mmp13*)	–1.85	3.55	–3.17	1.70	2.41
^∗^Defensin alpha 5 (*Defa5*)		3.31	–3.36	1.83	2.45
Rh-associated glycoprotein (*Rhag*)		3.18			2.86
Pro-platelet basic protein (*Ppbp*)		2.92	–4.08	2.14	4.13
^∗^Myeloperoxidase (*Mpo*)		2.86	–2.42	1.71	2.72
^∗^Hemogen (*Hemgn*)		2.63	–2.65		2.23
^∗^Neutrophilic granule protein (*Ngp*)		2.60	–1.62	1.57	
Rh blood group, D antigen (*Rhd*)		2.54	–2.49	1.84	1.95
^∗^Defensin RatNP-3 precursor (*RatNP-3b*)		2.50	–2.99		2.70
^∗^S100 calcium binding protein A8 (*S100a8*)		2.37	–2.22	2.10	1.74
^∗^Solute carrier family 4, member 1 (*Slc4a1*)		2.36	–1.93		2.26
Elastase, neutrophil expressed (*Elane*)		2.35	–3.00		2.93
^∗^S100 calcium binding protein A9 (*S100a9*)		2.35	–1.86	1.83	1.57
Eosinophil peroxidase (*Epx*)		2.30			
^∗^Alpha hemoglobin stabilizing protein (*Ahsp*)		2.29	–2.48		2.05
Multimerin 1 (*Mmrn1*)		2.24			1.90
^∗^Cathelicidin antimicrobial peptide (*Camp*)		2.16		1.36	1.75
Erythroblast membrane-associated protein (*Ermap*)		2.10			2.08
ATP binding cassette subfam. A member 13 (*Abca13*)		2.04			1.66
Kell blood group, metallo-endopeptidase (*Kel*)		2.00			2.47
Kruppel like factor 1 (*Klf1*)		2.00		1.52	1.75
Interleukin-23 receptor-like (*LOC103690079*)		1.92	–2.74		
Nuclear factor, erythroid 2 (*Nfe2*)		1.87			1.99
Roundabout homolog 1 *(Robo-1)*		1.82		2.13	2.09
5′-aminolevulinate synthase 2 (*Alas2*)		1.75	–1.99		1.53
T-cell Ig and mucin domain containing 2 (*Timd2*)		1.71		3.25	3.08
Hemoglobin, alpha 2 (*Hba2*)		1.68	–2.03		1.28
Ficolin B (*Fcnb*)		1.65		2.71	2.70
Hemoglobin, alpha 1 (*Hba1*)	–1.11	1.61	–1.89		1.17
Beta globin minor gene (*LOC100134871*)		1.58	–1.66		1.06
Aquaporin 1 (*Aqp1*)		1.54			1.57
Hemoglobin subunit beta (*Hbb*)	–1.14	1.49	–1.66		
Erythrocyte membrane protein band 4.2 (*Epb42*)		1.45			1.31
Hemoglobin, beta adult major chain (*Hbb-b1*)		1.30	–1.89		1.20
Integrin subunit alpha 2b (*Itga2b*)		1.12			1.23
AABR07043748.1 (unmapped)		–1.47			
Solute carrier family 17 member 7 (*Slc17a7, Vglut1*)		–2.16		–2.57	–2.97
Guanylate binding protein family member 6 (*Gbp6*)		–4.50	5.56	–3.73	–4.31
AABR07043200.1 (uncharacterized)		–6.33		–6.47	–6.22

*Also listed are those additionally selected by other contrasts involving injured animals: ±SCI(0), ±pim(st/SCI), ±st/SCI(0) and ±SCI(st). Quantities are log_2_(FC). Selection was made with edgeR analysis according to the criteria abs(log_2_(FC)) > 1 and FDR < 0.05. Genes marked by ^∗^ meet the stricter criterion FDR < 0.00001. Positive numbers indicate relative upregulation by injury, stimulation, pimozide or stimulation plus injury; negative numbers represent relative downregulation.*

The effect of stimulation on injury could also be shown less directly, by comparing genes flagged as altered by stimulation plus injury, in contrast ±st/SCI(0), with those flagged by injury alone, in contrast ±SCI(0). Some genes (*n* = 315) showed altered transcription after stimulation plus injury, in contrast ±st/SCI(0), but were not affected by injury alone, in contrast ±SCI(0). Most genes (*n* = 835) were flagged by both contrasts, and some (*n* = 147) were flagged by ±SCI(0) but not ±st/SCI(0). As seen in Table 2, of the 43 genes selected by contrast ±st(SCI), 15 were flagged by ±st/SCI(0) but not by ±SCI(0), 3 were flagged by ±SCI(0) but not by ±st/SCI(0) and one gene (*Mmmp13*) was flagged by both contrasts. This numerical imbalance confirms that stimulation caused changes in transcription mainly in genes that were unaffected by injury alone.

### Effect of Pimozide on the Response to Stimulation

Pimozide significantly altered 58% of the genes affected by stimulation after injury. Despite the possibility of bias for increases over decreases described above and the low sample number (*n* = 2 rats), genes flagged in contrast ±pim(st/SCI) showed a consistent direction of effect on genes flagged in contrast ±st(SCI) (Table 2). That is, upregulation was reversed in 24 genes and downregulation was reversed in one gene.

### Effect of Injury on the Response to Stimulation

Among rats that received stimulation, comparison of injured with intact groups, in contrast ±SCI(st), yielded further insight. A large number (*n* = 1374) of genes was flagged by ±SCI(st), most of which (36 of 43) were also flagged by contrasting stimulation with no stimulation, ±st(SCI) (Table 2). Injury thus profoundly alters the response to stimulation. Confirming this conclusion, the transcriptional response of intact rats to stimulation, as determined by contrast ±st(0), yielded a list of 28 flagged genes (Table 3), none of which was among those significantly affected by stimulation in injured rats [±st(SCI)]. Many genes flagged by ±st(0) were not protein-coding: 10 were genes for small RNAs. Unlike in injured rats, significant effects of stimulation in intact rats were in the same direction as the effects (when present) of injury and pimozide (Table 3).

**TABLE 3:** Genes selected by contrast ±st(0) (effect of stimulation in non-injured rats).

**Gene name**	**Transcript type**	**±st(0)**	**±pim(st/SCI)**	**±SCI(0)**
AABR07007730.1 (unmapped)	Processed_pseudogene	6.01		6.87
Kallikrein B1 (*Klkb1*)	Protein_coding	5.73		6.22
Rn50_X_0694.1 (unmapped)	Processed_pseudogene	5.70		
Similar to Tpi1 protein (*RGD1563601*)	Processed_pseudogene	3.77		
AABR07021745.1 (unmapped)	Small nuclear RNA	2.05	3.87	
Prothymosin alpha (*Ptma*)	Protein_coding	1.99		
AC127784.3 (unmapped)	Small nucleolar RNA	1.79	1.94	
Neutrophil immunoglobulin-like receptor 1 (*Nilr1*)	Protein_coding	1.77		3.45
AC129753.3 (unmapped)	Small nucleolar RNA	1.73	2.84	
Leukocyte immunoglobulin-like receptor (*Lilrb3*l)	Protein coding	1.71		2.99
AABR07067449.1 (unmapped)	Small nucleolar RNA	1.51		
AC118113.1 (unmapped)	Small nucleolar RNA	1.46		
AC112093.1 (unmapped)	Small nucleolar RNA	1.25		
AC097575.3 (unmapped)	Small nuclear RNA	1.24	1.58	
AABR07005613.1 (unmapped)	Miscellaneous_RNA	1.22		
AABR07050379.1 (unmapped)	Small nuclear RNA	1.22	2.02	
AABR07072283.4 (unmapped)	Small Cajal body-specific RNA	1.18		
Flavin containing monooxygenase 3 (*Fmo3*)	Protein coding	1.15		
Ribosomal_L22 domain containing protein (*RGD1359290*)	Protein coding	1.10		
AABR07036645.1 (unmapped)	Small nucleolar RNA	1.08	1.39	
AC094643.2 (unmapped)	Protein coding	–1.02		
ATPase subunit 8 (*mt-Atp8*)	Protein coding	–1.04		
Early growth response 1 (*Egr1*)	Protein coding	–1.07		
Cytochrome b (*mt-Cyb*)	Protein coding	–1.12		
NADH dehydrogenase subunit 2 (*mt-Nd2*)	Protein coding	–1.20		
NADH dehydrogenase subunit 4L (*mt-Nd4l*)	Protein coding	–1.52		
TNF receptor superfamily member 14 (*Tnfrsf14*)	Protein coding	–2.03		
Major intrinsic protein of lens fiber (*Mip*)	Protein coding	–3.01		

*Their transcript type is also listed shown. Quantities shown are log_2_(FC), as in Table 2. Genes that additionally showed significant effects of pimozide [±pim(st/SCI)] or untreated injury [±SCI(0)] are indicated in the adjacent columns.*

### Genes of Interest

**FIGURE 1:**
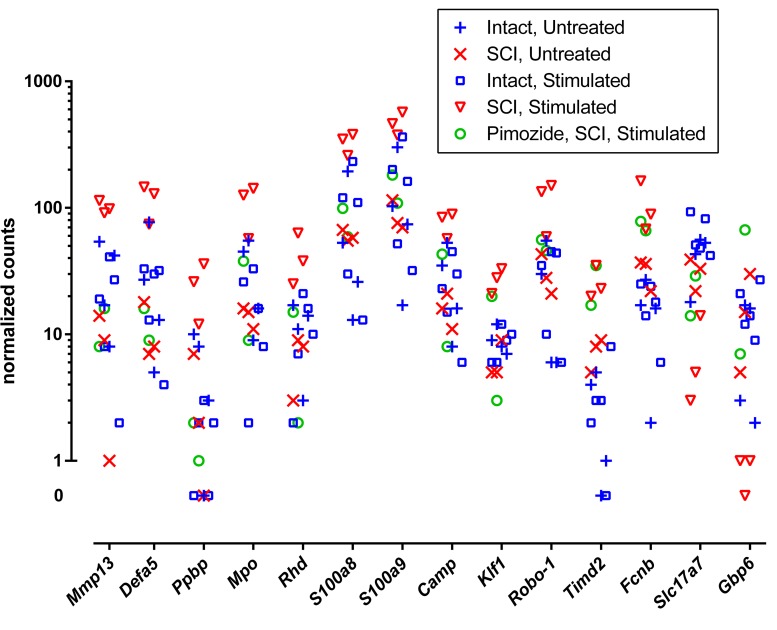
Normalized RNA molecular counts from individual rats for 14 characterized genes identified as prominently up-regulated (*n* = 12) or down-regulated (*n* = 2) by stimulation. The included genes were those flagged by all three contrasts ±st(SCI), ±SCI(st) and ±st/SCI(0), as listed in Table 2. The vertical axis is scaled logarithmically and log zero values (equal to –∞) are presented separately off-scale.

Individual genes of greatest interest for understanding the effect of NRM stimulation on SCI are those flagged by all of the three contrasts that involved stimulation (without pimozide) in injury: ±st(SCI), ±SCI(st) and ±st/SCI(0). Twelve of these genes were increased by stimulation: *Mmp13*, *Defa5*, *Ppbp*, *Mpo*, *Rhd*, *S100a8*, *S100a9*, *Camp*, *Klf1*, *Robo-1*, *Timd2*, and *Fcnb*. Of these 12 genes, 7 also passed the considerably more restrictive filtering criterion of FDR < 0.00001, and all except one of these (*Camp*) was reversed by pimozide (Table 2). Three genes that were flagged in these three contrasts were decreased by stimulation: *Slc17a7*, *Gbp6* and the uncharacterized *AABR07043200.1*, of which only the decrease in *Gbp6* was reversed by pimozide. Figure 1 shows the normalized counts for all 5 experimental conditions of the key characterized genes that were upregulated or down-regulated.

Few sRNA and miRNA genes underwent significantly altered transcription under any experimental condition. *Mir100* was down-regulated in the contrasts ±SCI(0) and ±SCI(st). *Mir3593* was down-regulated in the contrast ±SCI(st). Finally *Mir6326*, *Mir675*, and *Mir21* were up-regulated in the contrast ±SCI(st).

### Inferred Pathways and Biological Processes

Injury primarily increased inflammatory and immune transcripts and decreased those related to lipid and cholesterol synthesis and neuronal signaling, confirming previous reports (Bareyre and Schwab, 2003; Chamankhah et al., 2013; Shi et al., 2017). This was seen in the top canonical pathways determined with Ingenuity Pathway Analysis (Table 4) and in DAVID gene ontologies (biological process) for the contrast ±SCI(0) (some of which are listed in Table 5). Stimulation after injury had a major effect on pathways concerned with defense responses to lipopolysaccharides and various microorganisms (fungi, bacteria, yeast, viruses) and related inflammatory and immune responses (Tables 4, 5). Many of these same pathways were among the principal targets of pimozide. Also prominent among the pathways affected by stimulation in injury were erythrocyte development, oxygen transport and heme biosynthesis (Tables 4, 5). On the other hand, stimulation without injury, contrast ±st(0), had its largest effects on the canonical pathways for oxidative phosphorylation and mitochondrial dysfunction, with little influence on inflammatory and immune processes (Table 4). The DAVID gene ontologies (biological process) for contrast ±st(0) found that only two terms reached (weak) significance: “response to hyperoxia” and “regulation of apoptotic process.”

**TABLE 4:** Top canonical pathways indicated by IPA for the effects of injury alone [±SCI(0)], of stimulation after injury [±st(SCI)], stimulation on intact rats [±st(0)], and of pimozide on injured, stimulated rats [±pim(st/SCI)].

**Pathway**	***p*-Value**	**Overlap**
**±SCI(0) up-regulated**		
EIF2 signaling	4.59E−27	35.3% 78/221
TREM1 signaling	1.98E−16	45.3% 34/75
Role of macrophages, fibroblasts, and endothelial cells in rheumatoid arthritis	1.90E−14	23.6% 73/309
Fc receptor-mediated phagocytosis in macrophages and monocytes	7.15E−14	37.6% 35/93
Role of pattern recognition receptors in recognition of bacteria and viruses	1.75E−13	31.4% 43/137
**±SCI(0) down-regulated**		
Superpathway of cholesterol biosynthesis	9.06E−18	60.7% 17/28
Cholesterol biosynthesis I	1.56E−12	76.9% 10/13
Cholesterol biosynthesis II (via 24,25-dihydrolanosterol) 1.56E−12	1.56E−12	76.9% 10/13
Cholesterol biosynthesis III (via desmosterol)	1.56E−12	76.9% 10/13
Synaptic long term depression	1.43E−09	16.4% 24/146
**±st(SCI)**		
Role of IL-17A in psoriasis	2.13E−04	15.4% 2/13
Osteoarthritis pathway	5.34E−03	1.4% 3/211
Tetrapyrrole biosynthesis II	8.40E−03	20.0% 1/5
Heme biosynthesis II	1.51E−02	11.1% 1/9
Atherosclerosis signaling	1.94E−02	1.6% 2/127
**±st(0)**	**±st(SCI)**	**±st(SCI)**
Oxidative phosphorylation	1.21E−11	7.3% 8/109
Mitochondrial dysfunction	4.54E−10	4.7% 8/171
Sirtuin signaling pathway	9.91E−06	2.1% 6/291
Glycine degradation (creatine biosynthesis)	3.46E−03	50.0% 1/2
MSP-RON signaling pathway	6.92E−03	2.8% 2/72
**±pim(st/SCI)**		
Role of IL-17A in psoriasis	2.38E−04	15.4% 2/13
G protein signaling mediated by tubby	1.48E−03	6.2% 2/32
Tec kinase signaling	3.41E−03	1.8% 3/170
RhoGDI signaling	3.82E−03	1.7% 3/177
Agranulocyte adhesion and diapedesis	4.72E−03	1.6% 3/191

**TABLE 5:** Gene Ontology terms (biological process, GOTERM_BP_DIRECT) found to be enriched in by stimulation after injury [±st(SCI)] or its modification by pimozide [±pim(st/SCI)].

**Term**	**GO number**	**±st(SCI)**	**±pim(st/SCI)**	**±SCI(0)**
Defense response to fungus	0050832	6.52E−08	6.74E−08	
Erythrocyte development	0048821	1.60E−07	5.81E−04	
Oxygen transport	0015671	4.25E−06	6.20E−06	
Leukocyte migration involved in inflammatory response	0002523	2.02E−04	1.86E−04	
Defense response to bacterium	0042742	3.44E−04	3.83E−03	1.62E−02
Negative regulation of growth of symbiont in host	0044130	3.59E−04	1.33E−02	
Hydrogen peroxide catabolic process	0042744	3.61E−04	3.31E−04	
Killing of cells of other organism	0031640	4.36E−04	5.04E−04	
Ammonium transport	0015696	2.69E−03		
Ammonium transmembrane transport	0072488	4.14E−03		
Response to lipopolysaccharide	0032496	6.48E−03	7.80E−04	1.98E−09
Chronic inflammatory response	0002544	7.43E−03		
Cellular oxidant detoxification	0098869	8.26E−03	8.18E−03	
Response to yeast	0001878	8.55E−03		
Response to hydrogen peroxide	0042542	1.02E−02	1.08E−02	
Defense response to Gram-positive bacterium	0050830	1.55E−02		2.67E−07
Innate immune response	0045087	3.20E−02	9.36E−05	1.86E−13
Antibacterial humoral response	0019731	3.23E−02		
Response to virus	0009615		7.13E−04	4.20E−04
Defense response to virus	0051607		3.64E−03	1.07E−08

*Terms were obtained with DAVID 6.8. Those additionally enriched by injury in untreated rats [±SCI(0)] are also listed. The input data for DAVID came from gene lists containing both up-regulated and down-regulated genes that met the EdgeR criteria abs(log_2_(FC)) > 1 and FDR < 0.05. Values shown are Benjamini probabilities, which were applied to the enrichment criterion *p* < 0.05.*

### Resemblances of Expression Patterns to Perturbation With Drug Classes

The L1000 gene expression database of the CLUE software platform (clue.io) (Subramanian et al., 2017) was used to ascertain drug classes whose actions on cell cultures resembled to some quantifiable degree the effect of NRM stimulation on SCI. Human orthologs of the rat genes flagged in the two EdgeR contrasts ±st(SCI) and ±pim(st/SCI) were obtained from the Ensembl 2018 website (Zerbino et al., 2018). From the contrast ±st(SCI), which had flagged 43 rat genes, 37 human orthologous genes could be identified, and from the contrast ±pim(st/SCI), which originally yielded 81 rat genes, 65 human orthologous genes were identified (Supplementary Table 1). These two human gene lists were input to the CLUE Query tool to probe the database connectivity map, constructed from genetic changes in various human cell culture lines in response to diverse drugs. The classes of perturbagens (drugs) whose expression signatures most closely resembled those obtained in the experiments are listed in Table 6. The similarity score for the ±pim(st/SCI) contrast was reversed so that the absence of the drug pimozide was designated as the positive effect. High similarity (>98%) was reached in both contrasts by four perturbagen classes: PPAR agonists, angiotensin receptor antagonists, glucocorticoid receptor agonists, and nucleoporin loss of function (LOF).

**TABLE 6:** Perturbagens (pharmacologic and genetic) whose expression signatures most closely resemble the effect of stimulation after injury and its modification by pimozide.

**Drug class**	**% similarity**	**−(% similarity)**
	**±st(SCI)**	**±pim(st/SCI)**
PPAR receptor agonist	99.78	99.97
Nucleoporin LOF	99.59	99.73
Phospholipases GOF	99.46	81.78
Structural maintenance of chromosomes proteins LOF	99.40	<80%
Angiotensin receptor antagonist	99.02	99.22
Wnt family GOF	98.55	96.27
SRC inhibitor	98.53	<80%
Potassium channel blocker	98.37	<80%
Glucocorticoid receptor agonist	98.24	98.48
Imidazoline ligand	97.9	<80%
Bacterial DNA gyrase inhibitor	97.87	<80%
SRY LOF	97.61	93.55
NFkB pathway inhibitor	97.14	87.87
HMGCR inhibitor	96.44	<80%
FOS transcription factor family GOF	96.18	99.8
Heat shock 70 kDa proteins LOF	95.71	<80%
Proteasome pathway LOF	95.30	<80%
NKL subclass homeoboxes and pseudogenes LOF	94.86	<80%
Serine proteases GOF	94.59	98.28
Na-K-Cl transporter inhibitor	94.5	88.32

*The genes flagged as affected by stimulation in injury [contrast ±st(SCI)], as listed in Table 2, were converted to their human equivalents, if known, and fed to the Query function of the CLUE software platform (clue.io) (Subramanian et al., 2017). The genes flagged as affected by pimozide in rats that were both stimulated and injured [contrast ±pim(st/SCI)] were similarly processed, but their effects were reversed (i.e., similarity scores were multiplied by −1). GOF, genetic gain of function; LOF, genetic loss of function.*

## Discussion

### General Observations and Technical Considerations

The effect of the NRM, or indeed of any brainstem system, on transcription in the spinal cord has not previously been reported. Here we show that transcriptional changes occurred in the first 2 h of NRM stimulation in a small fraction of the total genome. A longer period of stimulation probably could have produced more changes, as seen with the 3-day injury period and even with the 3-h presence of pimozide. The effect of the NRM stimulation differed completely between injured and normal spinal cord. The changes due to stimulation were for the most part not reversals (or enhancements) of those caused by injury alone, and thus implicate a distinct set of genes. In SCI, stimulation affected primarily transcription in genes with defensive immune functions. Other notable changes were in genes involved in erythrocyte development, such as constituent molecules of hemoglobin. There was a paucity of neuron-specific effects. These general findings lead to a somewhat revised and more complex model of the NRM’s repair effect, with defensive and protective processes preceding (and perhaps permitting or evoking) the regenerative benefits of stimulation.

The validity of the findings and their interpretation must take into account the low numbers in each group. Low numbers are not uncommon in RNAseq studies, typically imposed by costs and by other external factors. A study of biological replicate numbers with various DGE software applications (Schurch et al., 2016), found that EdgeR, DESeq2, and baySeq can all produce satisfactory and mutually consistent conclusions with as few as three replicates, given a < 5% false discovery rate and a fold change of ±50% [or abs(log2(FC)) > 0.58]. Here, we applied a more restrictive criterion for fold change of ±100% [abs(log_2_(FC)) > 1] along with the < 5% false positive rate, and all groups except pimozide treatment consisted of > = 3 rats. Further strengthening the conclusions, the core list of altered genes (Figure 1, see section “Genes of Interest”) was derived from three paired contrasts that utilized all but two of the studied animals (*n* = 15). Thus the early changes in gene transcription found with NRM stimulation in injured animals can be specified with reasonable certainty. However, the findings strictly apply to female rats only, under conditions of treatment with buprenorphine, gentamycin and halothane, and the possibility remains of different genes emerging from contrasts made under other conditions or in other types of subject.

Pimozide is an antipsychotic drug that acts as an antagonist with a high affinity for both 5-HT7 receptors and for dopamine D2 subtype receptors (Roth et al., 1994). In the two subjects tested with pimozide, the drug consistently blocked and never enhanced the effects of stimulation in injury on individual genes. The descending pathways from the NRM are strongly serotonergic whereas dopaminergic projections are not prominent in the spinal cord and their cell bodies are distant to the site of stimulation (van Dijken et al., 1996). Thus the results with pimozide suggest that the effect of NRM stimulation after SCI was mediated in many genes by serotonin release, while a role for dopamine release is unlikely. The relatively rapid effect of pimozide in reversing gene expression induced by 2 h of NRM stimulation in SCI is likely to have occurred at the level of post-synaptic 5-HT7 receptors in the spinal cord. These receptors have been implicated in anti-inflammation, repair, and neuroplasticity at various CNS sites including the spinal cord (Kvachnina et al., 2005; de las Casas-Engel et al., 2013; Carballosa-Gonzalez et al., 2014; Di Pilato et al., 2014; Volpicelli et al., 2014; Fields and Mitchell, 2015). However, an action of pimozide at other CNS sites or other organs, which then indirectly causes a block of the influence of NRM stimulation on RNA synthesis in the spinal cord appears unlikely.

Measurements made after the 2 h time window of stimulation or after 3 days of injury represent snapshots of dynamic processes, which may include transient or long-lasting or even multiphasic changes in messenger RNA level. Changes in transcription rate are thought to determine the direction of most effects, because degradation rates and post-transcriptional processing are constant for most individual genes (Rabani et al., 2011, 2014), although inflammatory and immune signaling genes and targets of NFκB signaling are more likely to show modulated degradation (Rabani et al., 2011). The half-life of decay is typically slower than the transcription rate, which suggests the possibility of bias in favor of detecting increases over decreases. The half-life of messenger RNA in mammalian cell cultures averages 7–9 h, with a lower median, e.g., 4–6 h, and regulators of transcription and signal transduction tend to have shorter half-lives (Friedel et al., 2009; Schwanhausser et al., 2011). Therefore, given typical half-lives, the finding that 94% of changes were increases after the 3 day post-injury period probably reflects true differences in the direction of transcription rates. However, the finding that 91% of changes after the 2 h stimulation window were increases is alternatively explained by the slowness of decay.

### Background Studies

This work is part of a series exploring the central control of endogenous repair mechanisms. It is proposed that the various brainstem raphé nuclei, including the NRM, form the central links of feedback loops controlling repair. In our model, raphé neurons receive sensory, chemical and central-state signals indicative of recent injury (e.g., pain, vestibular input, hypothermia, circulating cytokines). Their evoked electrical activity then causes axonal release of serotonin and co-release of neuropeptide transmitters from terminals that, collectively, innervate almost the entire CNS (Molliver, 1987; Hornung, 2003). Trophic and protective cellular effects can be produced by these various neuropeptides: thyrotropin releasing hormone (Daimon et al., 2013), substance P (Jiang et al., 2012; Kim et al., 2015), galanin (Hobson et al., 2008), met-enkephalin (Narita et al., 2006; Campbell et al., 2012) as well as by serotonin (Azmitia, 2007; Trakhtenberg and Goldberg, 2012), all acting through non-synaptic volume transmission (Ridet et al., 1993; Agnati et al., 1995; Borroto-Escuela et al., 2015). As in the response to infection which is frequently concurrent with injury, an endogenous repair system from the perspective of natural selection should promiscuously exploit (facilitate or depress) multiple molecular and cellular mechanisms.

The main experimental evidence for this model is that some days or weeks of stimulation in a raphé nucleus starting in the first days following incomplete contusional SCI or fluid-pressure TBI or induction of MS-like signs was found to improve behavioral signs over several weeks (Hentall and Gonzalez, 2012; Carballosa Gonzalez et al., 2013; Madsen et al., 2017). Concomitant histological changes seen at chronic endpoints following stimulation include increased myelination and serotonergic innervation around the injury after T8 SCI (Hentall and Gonzalez, 2012), increased calcitonin gene related peptide after C5 SCI (Vitores et al., 2018), restored volume loss in neocortex after TBI (Carballosa Gonzalez et al., 2013), and lessened cellular pathology and cytokine production in experimental autoimmune encephalomyelitis (Madsen et al., 2017).

The mechanisms leading to these long-term changes are likely to involve complexly interwoven cellular and molecular processes. To better understand the proximate causes of improved recovery with NRM stimulation, this and some other studies have focused on more acute, post-injury endpoints and shorter stimulation periods. An immunostaining study of 3-day C5 injury after 2 days of NRM stimulation showed changes in numbers of inflammatory cell types and a transition from neural precursors to radial glia that facilitate differentiation (Jermakowicz et al., 2019). In rats with 3-day T8 contusions, 2 h of NRM stimulation, as in the present protocol, restored normal levels of injury-depleted cAMP, phosphorylated cAMP response binding element (pCREB), and pPKA (Carballosa-Gonzalez et al., 2014). The same study showed that NRM stimulation had no significant effects on levels of cAMP, pPKA or pCREB in intact animals, but pimozide in intact animals with or without stimulation lowered cAMP to injured levels. Reversal of cAMP depletion has also been shown in the neocortex and hippocampus of rats with 3-day old TBI following 3 days of electrical stimulation in the median raphé nucleus (Carballosa Gonzalez et al., 2013).

### Interpretation of Main Findings

Cyclic AMP can promote axonal regeneration following SCI via elevated pPKA and pCREB, which subsequently increases expression of various trophic genes, although a non-genetic route via pPKA can also have beneficial effects on the cytoskeleton via the small G protein, Rho (Lehmann et al., 1999; Hannila and Filbin, 2008). Given the previously demonstrated NRM-evoked recovery of cAMP after T8 SCI (Carballosa-Gonzalez et al., 2014), it was unsurprising that transcriptional effects of stimulation were seen. However, it was not expected that the main increases would be in defensive and pro-inflammatory genes, for example, *Ctsg*, *Np4*, *RatNP-3b*, *Defa5*, *Elane*, *Mpo*, *S100a8*, *S100a9*, and *Ppbp*. The proteins S100A8 and S100A9 are frequently combined as the antimicrobial dimer calprotectin (Striz and Trebichavsky, 2004). They probably arrive in the injured spinal cord as constituents of myeloid cells, particularly neutrophils (Fleming et al., 2006; Mitchell et al., 2008), which are thought to be deleterious to recovery (McCreedy et al., 2018), although S100A8 and S100A9 can also be induced in macrophages and microglia by neuropathic states (Abe et al., 1999). While there is some indirect evidence that S100A9 promotes recovery from SCI (Roet et al., 2013), but pro-inflammatory effects are more likely the dominant initial action.

Other genes upregulated by stimulation in SCI may also have deleterious effects. *Mpo* has been shown by gene knockout to exacerbate secondary injury after SCI (Kubota et al., 2012). *Ctsg* is a pronociceptive mediator in the spinal cord (Liu et al., 2015), as is carbonic anhydrases (gene *Car1*) (Asiedu et al., 2010). Neutrophil elastase, which is released by activated neutrophils, is reported to be a key mediator of secondary pathogenesis in SCI (Semple et al., 2015). On the other hand, matrix metalloproteinase 13 is a matrix-degrading enzyme released from monocytes that is important in functional recovery after SCI (Shechter et al., 2011). Of the several microRNAs tagged in these studies, *Mir21*, which was upregulated in the contrast ±SCI(st), is of greatest interest. *Mir21* is increased by exercise after SCI and improves recovery in rats, probably by regulating the pro-regenerative PTEN/mTOR pathway, since it lowers PTEN messenger RNA and raises mTOR messenger RNA (Liu et al., 2012). Few of the genes affected by stimulation after SCI (Table 2) are particularly associated with the CNS. One exception was the downregulated solute carrier family 17 member 7 (*Slc17a7*), also known as *Vglut1*, whose protein transports glutamate into synaptic vesicles (Shigeri et al., 2004); its levels in the spinal cord are reported to be decreased after peripheral nerve damage (Rotterman et al., 2014; Wang et al., 2016). Acute decreases in *Vglut1* (*Slc17a7*) with stimulation, as observed here, could lead to lower net glutamate release, which is likely to reduce damage due to over-stimulation of neurons (Krzyzanowska et al., 2014), although recovery ultimately requires restoration of *Vglut1* (Burnside et al., 2018). A second exception was *Robo-1*, which was increased by stimulation, whose protein product an important player in pioneer longitudinal axon guidance (Kim et al., 2011).

Another interesting group of genes unexpectedly upregulated by stimulation in injury is related to erythrocyte development and oxygen transport: *Klf1*, *Rhd*, *Rhag*, *Hbb*, *Hba*, *Hba2*, *Hbb-b1*. The last four produce components of hemoglobin, which is constitutively expressed in the brain and spinal cord (Biagioli et al., 2009; Richter et al., 2009; Russo et al., 2013), and may act protectively by sequestering oxygen and free radicals (Xie and Yang, 2016). Indeed, “cellular oxidant detoxification” also featured among biological processes enriched by stimulation after SCI (Table 5). The key regulator of erythropoiesis, erythropoietin (EPO), is known to promote recovery and regeneration in SCI (Gassmann et al., 2003; Matis and Birbilis, 2009). There were no significant changes in expression of the *Epo* gene in this study, but it is possible that the increase in erythropoietic genes was due to increased erythropoiten release (Hasselblatt et al., 2006). Both the protein and *Epo* messenger RNA are reported to be increased by serotonin in the mouse hippocampus (Choi and Son, 2013). Further study of how erythropoiten synthesis and release responds at different time points post-injury and during NRM stimulation appears to be warranted.

The prevalence of altered transcription in exogenous cells of the injured spinal cord requires further experimental investigation. Among various possibilities to test, arriving myeloid cells such as neutrophils could be more attracted to the spinal cord’s injury site after they have been altered in some way by NRM stimulation. Alternatively, transcription in exogenous cells, once arrived, could be influenced (perhaps toward a pro-regeneration phenotype) by the NRM stimulation or by local sequelae of that stimulation. Classic synapses are not necessary for the effects of the NRM stimulation, since both serotonin and co-released neuropeptides participate in non-synaptic volume transmission in the spinal cord (Ridet et al., 1993). A growing body of evidence implicates serotonin in both neural and humoral aspects of immune control. Serotonin and its receptors are present on numerous cells of the adaptive and innate immune systems and have been implicated in macrophage activation (Jiang et al., 2009; Krabbe et al., 2012; Baganz and Blakely, 2013).

## Conclusion

The present study adds important new details to the general concept that the raphé system senses and alleviates stress or injury. The effects of NRM stimulation on gene transcription were very different in injured and intact animals, mirroring our prior study of the effects of NRM stimulation on levels of cAMP, PKA, and CREB (Carballosa-Gonzalez et al., 2014). The earliest changes in gene transcription brought about by NRM stimulation were seen to be confined mainly to defensive and erythropoietic functions. Whereas a week or more of NRM stimulation facilitates repair (Hentall and Burns, 2009; Hentall and Gonzalez, 2012; Madsen et al., 2017; Vitores et al., 2018), the shorter 2-h period used here intensified transcription of predominantly inflammatory genes. Although the products of these genes are often assumed to be harmful, immune responses cannot necessarily be simply parsed into harmful and beneficial processes (Doty et al., 2015; Li et al., 2018). Conceivably, the inflammatory effects of substances such as S100A8 and S100A9, however harmful they may be initially, are necessary preliminaries with net benefits to the overall repair process, and lead later to neural-specific effects. Consistent with these ideas, three of the four top perturbagen classes that resembled the effect of 2 h of stimulation (Table 6) are known to produce improvements in SCI: PPARs agonists (Park et al., 2007), angiotensin receptor antagonists (Guler et al., 2010) and glucocorticoid receptor agonists, although this last group’s benefits in SCI are more controversial (Akhtar et al., 2009). Furthermore, all three classes inactivate inflammatory pathways, such as those controlled by NF-κB (Bekhbat et al., 2017; Labandeira-Garcia et al., 2017; Villapol, 2018). The present study thus tends to confirm the therapeutic promise of these drug classes. The study also provides further support for interim DBS of the NRM or, more practically from the surgical standpoint, of its midbrain periaqueductal gray input, as a therapy for SCI that may ultimately be superior to single or combined drugs in its simplicity and safety of application and the range of signs and symptoms improved (Chari et al., 2017).

## Data Availability Statement

The datasets generated for this study can be found in the National Center for Biotechnology Information (NCBI), GEO Accession Number GSE133093.

## Ethics Statement

The animal study was reviewed and approved by the Institutional Animal Care and Use Committee of the University of Miami, Miller School of Medicine.

## Author Contributions

IH designed the experiments and wrote the manuscript. WJ managed the RNA sequencing and analysis. MC-G and AV performed animal surgery, stimulation, and tissue extraction.

## Conflict of Interest

The authors declare that the research was conducted in the absence of any commercial or financial relationships that could be construed as a potential conflict of interest.

## Footnotes

1 http://www.bioinformatics.babraham.ac.uk/projects/fastqc/

2 www.bioinformatics.babraham.ac.uk/projects/trim_galore

3 https://david.ncifcrf.gov/tools.jsp

## Abbreviations

cAMP: cyclic adenosine monophosphate
DAVID, Database for Annotation: Visualization and Integrated Discovery
DBS: deep brain stimulation
DGE: differential gene expression
EPO: erythropoietin
FC: fold change
MS: multiple sclerosis
NCBI: National Center for Biotechnology Information
NRM: nucleus raphé magnus
pCREB: phosphorylated cAMP response binding element
pim: pimozide
PPAR: peroxisome proliferator-activated receptor
pPKA: phosphorylated protein kinase A
SCI: spinal cord injury
st: stimulation
TBI: traumatic brain injury
