# Supplementary material for: Brainstem-Evoked Transcription of Defensive Genes After Spinal Cord Injury
Source: Front Cell Neurosci. 2019 Nov 19;13:510. doi: 10.3389/fncel.2019.00510 (PMC6877476; doi:10.3389/fncel.2019.00510)
Supplement: Supplementary file 1 [file Data_Sheet_1.DOCX]

Supplementary Figure 1. Overlap in the numbers of genes flagged with the three types of DGE software (EdgeR, DESeq2 and baySeq) for the six effects (contrasts) studied: injury alone, ±SCI(0); stimulation in injured rats, ±st(SCI); pimozide in stimulated and injured rats, ±pim(st/SCI); stimulation plus injury versus intact untreated rats, ±st/SCI(0); stimulation in intact rats, ±st(0); injury in stimulated rats, ±SCI(st). Selection criteria were abs(log_2_(FC)) >1 combined with FDR <0.05 for EdgeR, with padj <0.05 for DESeq2 and with FDR.DE<0.05 for baySeq. (FDR is false discovery rate and padj is adjusted probability.) The 32,494 transcripts with unique rat gene identification (Ensembl) numbers included 43 antisense, 3069 long intergenic noncoding RNA, 1,563 micro RNA, 522 miscellaneous RNA, 24 mitochondrial RNA, 718 processed pseudogenes, 72 processed transcripts, 22,019 protein coding, 794 pseudogenes, 39 ribozymes, 321 ribosomal RNA, 51 small Cajal body-specific RNA, 10 sense intronic, 1,646 small nucleolar RNA, 1,471 small RNA, 30 to be confirmed experimentally, 13 transcribed processed pseudogenes 117, unprocessed pseudogenes, and 43 untyped.
