# Supplementary material for: Brainstem-Evoked Transcription of Defensive Genes After Spinal Cord Injury
Source: Front Cell Neurosci. 2019 Nov 19;13:510. doi: 10.3389/fncel.2019.00510 (PMC6877476; doi:10.3389/fncel.2019.00510)
Supplement: Supplementary file 2 [file Table_1.DOCX]

Supplementary Table 1. Human genes orthologous to those flagged in contrasts of stimulation with no stimulation in injured rats ±st(SCI) and of pimozide with no pimozide in injured and stimulated rats ±pim(st/SCI). Significant contrasts are indicated by log_2_(FC); values are missing where the contrast was not significant. The first column contains the Ensembl rat gene number without the normally preceding ‘ENSRNOG000000’; ‘x’ indicates no corresponding human gene. Abbreviations: ‘pc’, protein-coding; ‘pseudo’, pseudogene; ‘Mt tRNA’, mitochondrial transfer tRNA; ‘snoRNA’, small nucleolar RNA; snRNA, small nuclear RNA; ‘misc RNA’ miscellaneous RNA.

| Gene number | RAT | HUMAN | ±st(SCI) | ±pim(st/SCI) | type |
| --- | --- | --- | --- | --- | --- |
| 167 | *Alas2* | *ALAS2* | 1.75 | -1.99 | pc |
| 335 | *Ermap* | *ERMAP* | 2.10 |  | pc |
| 542 | *Dnah8* | *DNAH8* |  | 1.99 | pc |
| 1187 | *Oasl* | *OASL* |  | 2.14 | pc |
| 1827 | *Masp1* | *MASP1* |  | 1.09 | pc |
| 1959 | *Mx1* | *MX1* |  | 2.21 | pc |
| 1963 | *Mx2* | *MX1* |  | 1.84 | pc |
| 2134 | *Gbp6* | *GBP6* | -4.50 | 5.56 | pc |
| 2820 | *Robo-1* | x | 1.82 |  | pc |
| 2829 | *Ppbp* | *PPBP* | 2.92 | -4.08 | pc |
| 3028 | *Dnah17* | *DNAH17* |  | 2.03 | pc |
| 3244 | *Ltc4s* | *LTC4S* |  | 1.91 | pc |
| 3443 | *Klf1* | *KLF1* | 2.00 |  | pc |
| 5116 | *Bcl2l1* | x |  | 1.94 | pseudo |
| 8310 | *Mpo* | *MPO* | 2.86 | -2.42 | pc |
| 8478 | *Mmp13* | *MMP13* | 3.55 | -3.17 | pc |
| 8536 | *Actc1* | *ACTC1* |  | 6.66 | pc |
| 8707 | *Epx* | *EPX* | 2.30 |  | pc |
| 9342 | *Fcnb* | *FCN2* | 1.65 |  | pc |
| 9436 | *Hemgn* | *HEMGN* | 2.63 | -2.65 | pc |
| 10698 | *Car1* | *CA1* | 3.78 | -2.49 | pc |
| 11175 | *Hnrnpa2b1* | *HNRNPA2B1* |  | -1.02 | pc |
| 11483 | *S100a9* | *S100A9* | 2.35 | -1.86 | pc |
| 11557 | *S100a8* | *S100A8* | 2.37 | -2.22 | pc |
| 11648 | *Aqp1* | *AQP1* | 1.54 |  | pc |
| 11649 | *Epb42* | *EPB42* | 1.45 |  | pc |
| 15541 | *Gnb3* | *GNB3* |  | -2.51 | pc |
| 15682 | *Kel* | *KEL* | 2.00 |  | pc |
| 17130 | *Rhd* | *RHD* | 2.54 | -2.49 | pc |
| 20165 | *Ahsp* | *AHSP* | 2.29 | -2.48 | pc |
| 20647 | *Ctsg* | *CTSG* | 3.90 | -2.26 | pc |
| 20650 | *Slc17a7* | *SLC17A7* | -2.16 |  | pc |
| 20733 | *Camp* | *CAMP* | 2.16 |  | pc |
| 20951 | *Slc4a1* | *SLC4A1* | 2.36 | -1.93 | pc |
| 21345 | *Timd2* | *HAVCR1* | 1.71 |  | pc |
| 22071 | *Itga2b* | *ITGA2B* | 1.12 |  | pc |
| 23733 | *RGD1560821* | x |  | 1.72 | pc |
| 24330 | *Ngp* | x | 2.60 | -1.62 | pc |
| 24986 | *Mmrn1* | *MMRN1* | 2.24 |  | pc |
| 25645 | *Abca13* | x | 2.04 |  | pc |
| 25959 | *Gp1ba* | *GP1BA* |  | -6.45 | pc |
| 28707 | *Np4* | *DEFA4* | 3.69 | -2.85 | pc |
| 28993 | *LOC102555453* | x |  | -1.42 | pc |
| 29115 | *RGD1564883* | x | 7.12 | -10.44 | pc |
| 29195 | *Uba7* | *UBA7* |  | 1.14 | pc |
| 29886 | *Hba-a1* | *HBA2* | 1.61 | -1.89 | pc |
| 30093 | *Defa5* | *DEFA4* | 3.31 | -3.36 | pc |
| 32274 | *AY172581.15* | x |  | 3.08 | Mt tRNA |
| 33685 | *Elane* | *ELANE* | 2.35 | -3.00 | pc |
| 33916 | *Rps21-ps1* | *RPS21* |  | 2.10 | pc |
| 34246 | *Rps27a-ps1* | *RPS27A* |  | 1.80 | pc |
| 36604 | *Ifit2* | *IFIT2* |  | 1.29 | pc |
| 36837 | *Nfe2* | *NFE2* | 1.87 |  | pc |
| 38135 | *RatNP-3b* | *DEFA4* | 2.50 | -2.99 | pc |
| 46151 | *Tubb1* | *TUBB1* |  | -2.14 | pc |
| 46621 | *AABR07043748.1* | *EIF5B* | -1.47 |  | pc |
| 47098 | *Hbb-b1* | *HBB* | 1.30 | -1.89 | pc |
| 47321 | *Hba-a2* | *HBA2* | 1.68 | -2.03 | pc |
| 49070 | *Rack1* | *RACK1* |  | 7.65 | pc |
| 49282 | *Oas2* | *OAS2* |  | 2.26 | pc |
| 49829 | *LOC103690079* | x | 1.92 | -2.74 | pc |
| 50121 | *Rhag* | *RHAG* | 3.18 |  | pc |
| 51492 | *AC135285.4* | *SNORA74A* |  | 3.77 | snoRNA |
| 52180 | *AABR07052780.3* | *RN7SL674P* |  | 2.65 | misc RNA |
| 52373 | *AABR07031718.1* | *VTRNA1-3* |  | 3.05 | misc RNA |
| 52617 | *AABR07052881.1* | *SNORD67* |  | 2.61 | snoRNA |
| 52741 | *AC134024.2* | *SNORA21* |  | 2.11 | snoRNA |
| 52742 | *AC097575.3* | *RNU4-1* |  | 1.58 | snRNA |
| 52992 | *AABR07019037.1* | x |  | 5.66 | snRNA |
| 53452 | *LOC100361457* | x |  | 1.76 | pc |
| 53764 | *AC099450.2* | *SNORD33* |  | 2.67 | snoRNA |
| 53902 | *AABR07072283.2* | *x* |  | 2.54 | misc RNA |
| 54091 | *AC094348.2* | *SNORA38B* |  | 1.56 | snoRNA |
| 54533 | *AABR07072025.1* | *SNORA57* |  | 1.23 | snoRNA |
| 54746 | *AABR07036645.1* | *SNORA49* |  | 1.39 | snoRNA |
| 55021 | *AABR07051357.1* | *RNU6ATAC* |  | 3.78 | snRNA |
| 55352 | *AABR07032119.1* | x |  | 2.00 | snRNA |
| 55465 | *AABR07071745.2* | *RF00091* |  | 2.60 | snoRNA |
| 55560 | *AC099450.3* | *SNORD32A* |  | 2.17 | snoRNA |
| 56054 | *AC129753.3* | *SNORD118* |  | 2.84 | snoRNA |
| 56608 | *AABR07060522.2* | *RF00019* |  | 3.58 | misc RNA |
| 56978 | *AABR07036375.1* | x |  | 1.59 | snoRNA |
| 57562 | *AABR07021745.1* | *RNU5E-1* |  | 3.87 | snRNA |
| 57838 | *AABR07050379.1* | x |  | 2.02 | snRNA |
| 58105 | *Hbb* | *HBB* | 1.49 | -1.66 | pc |
| 58276 | *AABR07043200.1* | x | -6.33 |  | snoRNA |
| 58269 | *AC127784.3* | *SNORD83B* |  | 1.94 | pc |
| 58881 | *AABR07067827.4* | *RF00554* |  | 1.25 | snoRNA |
| 58970 | *AABR07022185.1* | *RNU5E-4P* |  | 3.76 | snRNA |
| 58981 | *AABR07031683.1* | *SNORA13* |  | 2.09 | snoRNA |
| 59272 | *AC119459.4* | *RNU5E-1* |  | 3.69 | snRNA |
| 59280 | *AC119459.5* | x |  | 2.39 | snRNA |
| 59449 | *AABR07029907.3* | x |  | 2.48 | misc RNA |
| 60430 | *AABR07060522.3* | *RF00019* |  | 2.93 | misc RNA |
| 60522 | *AABR07046765.2* | *RN7SL4P* |  | 2.37 | misc RNA |
| 60810 | *AC114512.2* | x |  | 1.46 | snoRNA |
| 60816 | *AC136563.3* | *RF00554* |  | 1.28 | snoRNA |
| 61093 | *AC119459.6* | *RNU5E-1* |  | 2.81 | snRNA |
| 61299 | *LOC103694857* | *HBB* | 1.58 | -1.66 | pc |
